# Biological functions and applications of circRNAs—next generation of RNA-based therapy

**DOI:** 10.1093/jmcb/mjad031

**Published:** 2023-05-05

**Authors:** Meiling Sun, Yun Yang

**Affiliations:** Research and Development Department, Shanghai CirCode Biomedicine Co. Ltd, Shanghai, China; Research and Development Department, Shanghai CirCode Biomedicine Co. Ltd, Shanghai, China

**Keywords:** circular RNA, cap-independent translation, RNA-based therapy, biological function

## Introduction

Circular RNAs (circRNAs) are a unique class of single-stranded RNA molecules that are covalently closed and lack 5′ and 3′ ends. They were first discovered in eukaryotic cells >30 years ago, but technical limitations hindered the characterization of circRNAs until the 2010s. Recent advances in high-throughput RNA sequencing (RNA-seq), coupled with circRNA enrichment using RNase R treatment and new bioinformatic tools, have led to the discovery of a large number of circRNAs in various eukaryotic organisms, including mammals, insects, nematodes, plants, and yeast. Most circRNAs are generated through back-splicing of pre-mRNAs. Several studies showed that the expression levels of some circRNAs are significantly higher than that of their corresponding linear RNAs (Jeck et al., 2013; Salzman et al., 2013), indicating that circRNA biogenesis is tightly regulated. Although the mechanisms of circRNA biogenesis are still unclear, there are three regulatory models: (i) the RNA secondary structure mediates back-splicing through complementary sequences, such as Alu repeat sequences; (ii) RNA-binding proteins facilitate back-splicing, such as QKI; and (iii) the splicing intermediate lariat drives back-splicing.

Some studies showed that most circRNAs are non-functional ([Bibr bib26][Bibr bib26]), while several other studies showed that circRNAs can function as miRNA sponges, protein sponges, protein scaffolds, or translation templates. In addition, circRNAs are much more stable than linear RNAs in cells, making them a promising platform for RNA therapy. Here, we discuss the biological functions and therapeutic potential of circRNAs.

## Biological functions of circRNAs

Based on large datasets of high-throughput RNA-seq, thousands of circRNAs were identified in different types of cells. Most circRNAs are expressed at lower levels compared with their linear RNA isoforms from the same gene (Jeck et al., 2013; Salzman et al., 2013), and only a small fraction have been functionally characterized. Therefore, the biological functions of circRNAs are still debated.

**Figure 1:**
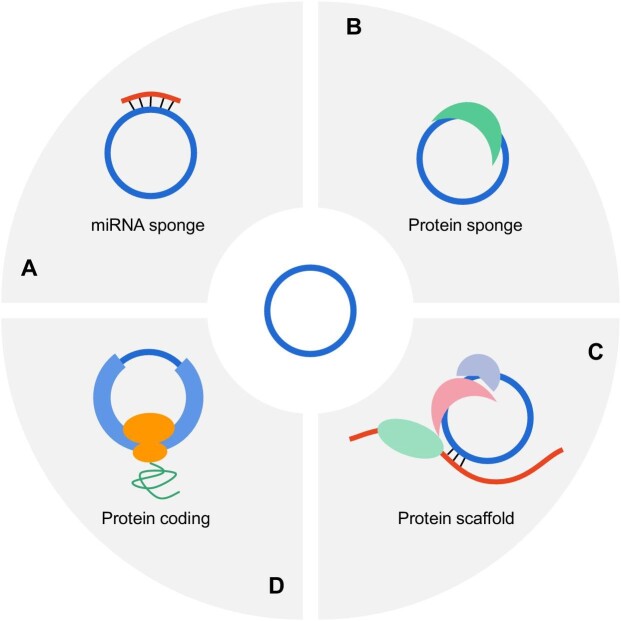
Biological functions of circRNAs. (**A**) CircRNAs can regulate miRNA levels as miRNA sponges and compete with target mRNAs for miRNA binding. Thus, the translation of target mRNAs is regulated. (**B**) CircRNAs can bind with RNA-binding proteins, affecting their functions and distributions. (**C**) CircRNAs can recruit multiple proteins to form complexes for special functions. (**D**) CircRNAs can encode functional proteins.

Most studies showed that circRNAs can function as miRNA sponges or decoys to regulate target mRNAs (Figure 1A). A well-characterized example is ciRS-7/CDR1as, a circRNA in human brain, that contains ∼70 conserved binding sites for miR-7 and functions as the ‘sponge’ of miR-7 (Hansen et al., 2013; Memczak et al., 2013). ciRS-7/CDR1as is highly and stably expressed in many tissues, especially the brain, and may regulate miR-7 in different tissues through different mechanisms (Kristensen et al., 2019). Knocking down ciRS-7 from the mouse genome downregulates the expression of miR-7 in the mouse brain, whereas other studies showed that ciRS-7 expression inhibits miR-7 expression. Interestingly, miR-7-targeting genes (such as *Fos*) are upregulated in ciRS-7 knockout mice and thus affect brain function. Other circ-RNAs, such as circHIPK3 and circBIRC6, were also reported to regulate miRNA function as miRNA sponges (see the review Kristensen et al., 2019 for more details).

Similarly, circ RNAs containing some protein-binding sites can interact with special proteins and thus function as protein sponges (Figure 1B). For example, circMbl is a circRNA generated from the gene encoding the splicing factor muscleblind (MBL) in *Drosophila melanogaster*. circMbl and its flanking introns contain conserved MBL-binding sites, which are strongly and specifically bound by MBL. The splicing factor MBL binds to its pre-mRNA to facilitate back-splicing. As feedback, the resulting circMbl can sequester MBL to promote linear splicing (Ashwal-Fluss et al., 2014). circPABPN1 competes with its cognate mRNA for the binding of HuR, reducing PABPN1 mRNA translation. In addition, circANRIL can bind to pescadillo homolog 1 (PES1) to impair exonuclease-mediated pre-rRNA processing and ribosome bio-genesis (see the review Kristensen et al., 2019 for more details). CircRNAs function as protein sponges to sequester the RNA-binding proteins in these cases. Interestingly, several antiviral proteins, such as NF90/NF110 and PKR, preferentially bind to circRNAs rather than their corresponding linear RNAs (Liu et al., 2019). Viral infection can trigger genome-wide degradation of circRNAs. Subsequently, NF90/NF110 and PKR can be rapidly released from circRNAs and then recognize viral double-stranded RNAs to initiate the innate immune response. In this case, circRNAs function as protein reservoirs to store the essential proteins for a rapid response.

Non-coding RNAs, including circRNAs, can function as protein scaffolds to assemble large RNA–protein complexes, such as ribosomal and spliceosomal RNAs (Figure 1C). For instance, circAmotl1 and circFoxo3 were reported to facilitate the colocalization of enzymes and their substrates to promote protein modification. Moreover, circFECR1 can recruit TET1 to the promoter region of its host gene to activate transcription (see the review Kristensen et al., 2019 for more details).

Since circRNAs lack the 5′-cap and 3′-poly(A) tail, they were considered non-coding RNAs. However, cap-independent translation, an alternative method that can initiate translation without a 5′-cap and 3′-poly(A) tail, is involved in the translation of some circRNAs (Figure 1D). This deepens our understanding of circRNA functions. Chen and Sarnow (1995) first reported that an *in vitro* synthesized circRNA containing a viral internal ribosome entry site (IRES) could be translated in an *in vitro* translation system (rabbit reticulocyte lysate). Twenty years later, Wang and Wang (2015) found that the circRNA comprising a viral IRES can be used to produce functional proteins in human living cells, indicating that circRNAs can serve as mRNAs. A subsequent study showed that N^6^-methyladenosine modification in circRNAs also drove their translation in a cap-independent fashion (Yang et al., 2017). Moreover, several groups found that endogenous cellular circRNAs can produce new proteins with different biological functions (Legnini et al., 2017; Pamudurti et al., 2017; Yang et al., 2018), suggesting that circRNAs may serve as the templates for protein translation to increase proteome diversity.

Recent studies have demonstrated that circRNAs play important roles in multiple diseases, such as cancer, cardiovascular disease, and neurodegenerative diseases (see the review Verduci et al., 2021 for more details). For example, circPVT1 was found overexpressed in head and neck squamous cell carcinoma, particularly in patients with TP53 mutations, and acted as an oncogene to modulate the expression of miR-497-5p and genes involved in the control of cell proliferation. Therefore, circRNAs are potential therapeutic targets for cancer and other diseases. Due to their high tissue specificity and stability in tissues and body fluids, they may also serve as new biomarkers for disease diagnosis and progression monitoring.

## Characteristics of circRNAs

**Figure 2:**
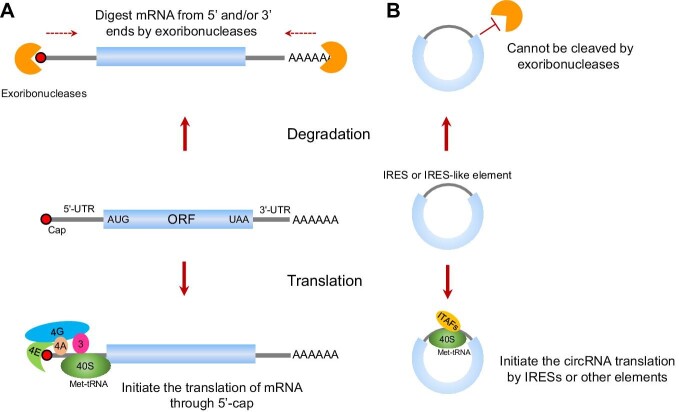
Comparison of circRNAs and linear mRNAs. (**A**) Each mRNA contains a 5′-cap and a 3′-poly(A) tail, and thus can be translated through cap-dependent translation. Most mRNAs are degraded by exonucleases from the 5′ and/or 3′ ends. (**B**) CircRNAs lack 5′ and 3′ ends, and thus are resistant to the degradation of exonucleases and can be translated in a cap-independent fashion.

CircRNAs are a type of single-stranded RNA molecule that is covalently closed and have unique features that distinguish them from mRNAs (Figure 2). First, unlike eukaryotic mRNAs, which typically consist of five regions (the 5′-cap, 5′-UTR, open reading frame, 3′-UTR, and 3′-poly(A) tail), circRNAs lack the 5′-cap and 3′-poly(A) tail. Second, most mRNAs are translated by ribosome recruitment to the 5′-cap, followed by ribosome scanning toward a start codon in eukaryotic cells. This process is called cap-dependent translation. Sometimes, mRNAs can also recruit ribosomes through IRES to promote translation. Nevertheless, circRNAs are translated in a cap-independent manner (Wang and Wang, 2015; Legnini et al., 2017; Pamudurti et al., 2017; Yang et al., 2018). In addition, the translation of circRNAs can be tissue-specific, depending on the special elements (such as IRESs) in circRNAs. Third, since mRNA turnover plays a key role in the control of gene expression, the mRNA degradation system can efficiently eliminate mRNAs in cells. Most mRNAs are degraded by 5′ exonucleases, which digest RNAs from the 5′ end, and 3′ exonucleases, which promote hydrolysis at the 3′ end. Instead, circRNAs can only be digested by several endonucleases (see the review Ren et al., 2022 for more details), which makes them more stable than mRNAs.

## Therapeutic potential of circRNAs

CircRNAs exhibit versatile functions, serving as mRNAs to produce proteins or acting as non-coding RNAs to regulate biological processes, and thus can be a promising platform for the development of nucleic acid therapy. CircRNA is more stable than mRNA, making it suitable for long-term protein expression or target regulation (Table 1). CircRNA is less immunogenic and safer than viral vector-based and DNA therapy (Table 1). These features make the circRNA platform an attractive option for developing the next generation of RNA-based therapy. In this context, we present several possibilities for circRNA therapy.

**Table 1:** **Comparison of circRNA therapy and other approaches**.

	Viral vector	DNA	mRNA	CircRNA
Expression duration	Years	Weeks	Days	Weeks
Delivery	Efficient transduction into postmitotic cells	The non-viral delivery system is inefficient, but its cytotoxicity and immunogenicity are generally low	The non-viral delivery system is inefficient, but its cytotoxicity and immunogenicity are generally low	The non-viral delivery system is inefficient, but its cytotoxicity and immunogenicity are generally low
Safety	May induce mutagenesis, with high cytotoxicity and immunogenicity	May induce mutagenesis	Do not induce mutagenesis	Do not induce mutagenesis
Manufacture	Cell-based production and high manufacturing cost	Easy to produce and manufacture cost-effectively	Easy to produce and manufacture cost-effectively	Easy to produce and manufacture cost-effectively
Limitation	It is not suitable for repeated doses because of its immunogenicity and the size of the cargo is limited	It is very difficult to transduce into postmitotic cells	It is unstable and not suitable for long-term expression	It is more stable than mRNA, but its expression duration depends on the life cycle of the host cell

**Figure 3:**
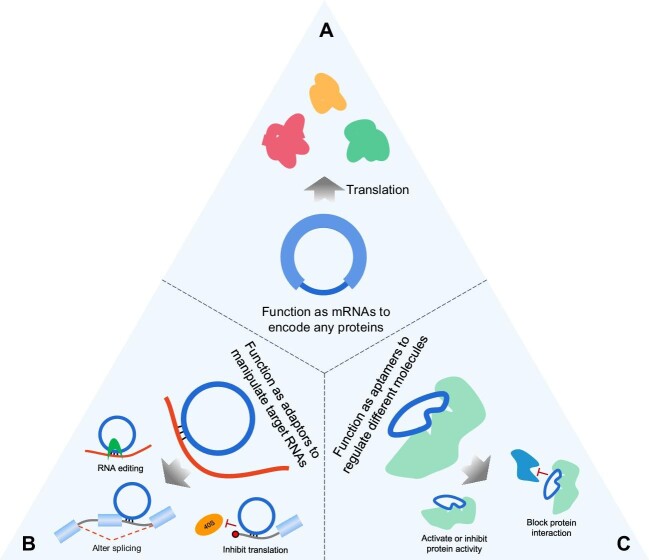
Therapeutic potential of circRNAs. (**A**) Circular mRNAs. IRES or other cap-independent translation initiation element and the optimized ORF can be designed and inserted into a circRNA. This circRNA can produce therapeutic proteins in the human body. (**B**) Circular adaptors. Base-pairing regions and/or protein-binding sites can be added to a circRNA to modulate gene expression. (**C**) Circular aptamers. Special aptamer sequences can be inserted into a circRNA to regulate protein function.

mRNAs direct cells to produce functional proteins through cellular translation machinery (ribosomes), and thus are ideal for vaccines, protein replacement therapeutic drugs, and other applications. However, mRNA stability and tissue-specific expression remain significant challenges. CircRNAs can overcome these issues, being more stable and translated in a cap-independent manner. For example, the *in vitro* synthesized circRNA that codes for hEPO protein can be robustly expressed for a long time in cells and mice (Wesselhoeft et al., 2019). Moreover, the circRNA vaccine can protect mice and rhesus macaques from SARS-CoV-2 infection by expressing the trimeric receptor-binding domain of the spike protein (Qu et al., 2022). Therefore, circRNAs have the potential to be a new drug modality for expressing therapeutic proteins or antigens *in vivo* (Figure 3A). Additionally, circRNAs can be designed to produce proteins in a tissue- or cell type-specific manner according to the expression levels of IRES *trans*-acting factors (Yang and Wang, 2019).

Furthermore, circRNAs can be used as antisense RNAs or adaptors to manipulate target RNA or DNA functions (Figure 3B). Pfafenrot et al. (2021) designed antisense circRNAs and oligos that are paired with the 5′-UTR of SARS-CoV-2 viral mRNAs to inhibit virus proliferation in cells. They found that the inhibition efficiency of antisense-circRNAs was better than that of the corresponding linear modified antisense oligos due to the high stability in cells (Pfafenrot et al., 2021). Additionally, circular and linear guide RNAs were used to recruit ADAR proteins to edit target mRNAs, and circular guide RNAs exhibited greater editing efficiency than linear guide RNAs (Katrekar et al., 2022; Yi et al., 2022). The high stability of circRNAs can increase the efficiency of circRNA-mediated regulation, making it an effective tool for manipulating target RNAs or DNAs.

RNAs can be designed as aptamers to bind different proteins or small molecules to regulate their functions. Aptamers are called chemical antibodies. However, linear RNA aptamers are rapidly degraded by exoribonucleases in cells. The Tornado (Twister-optimized RNA for durable overexpression) expression system was designed to produce circRNA-based aptamers, which are highly stable and achieve remarkably high expression levels in cells (Litke and Jaffrey, 2019). Thus, circRNAs can be used as aptamers with high stability and activity (Figure 3C).

## *In vitro* production of circRNAs

The increasing number of circRNA species discovered *in vivo* and the therapeutic potential of circRNAs have sparked a growing interest in strategies for RNA circularization *in vitro*. Various approaches have been developed for generating circRNAs *in vitro*, including chemical, enzymatic, and ribozyme strategies, which are summarized below (Table 2).

**Table 2:** **Summary of RNA circularization methods**.

Method	Advantages	Disadvantages
Chemical ligation	Effective for very small circRNAs (2–100 nucleotides)	Competition from intermolecular ligations and formation of 2′–5′ phosphodiester bonds
Enzymatic ligation by DNA ligase 1	Effective for larger circRNAs; ligation is sequence-specific; agnostic to terminal nucleotide identity; can produce exact sequence mimics	Competition from intermolecular ligations; requires optimization of the splint with each new substrate; higher amounts of the enzyme are needed due to low turnover
Enzymatic ligation by T4 RNA ligase 1	Effective for larger circRNAs; higher efficiency; splint not needed; easier to purify due to minimal reaction components; can produce exact sequence mimic	The efficiency of circularization is highly dependent on the structure of linear precursor; competition from intermolecular ligations; lower sequence specificity; strong nucleotide preference at 3′ and 5′ ends
Enzymatic ligation by T4 RNA ligase 2	Effective for larger circRNAs; greater substrate flexibility; splint not needed; agnostic to terminal nucleotide identity; can produce exact sequence mimic	The efficiency of circularization is highly dependent on the structure of linear precursor; competition from intermolecular ligations
PIE system with group I introns	Effective for various RNA sizes (100 nucleotides–5 kb); can be applied both *in vivo* and *in vitro*	CircRNAs retain portions of the exons from the native group I intron-containing genes; several splicing intermediates are produced as byproducts
PIE system with group II introns	It can be used to mimic an endogenous circRNA sequence precisely without any extraneous sequence; can be applied both *in vivo* and *in vitro*	Limited characterization
Hairpin ribozymes	Can generate high yields of small circRNAs (∼50–150 nucleotides) with sequence homogeneity	CircRNAs contain ribozyme sequences and cognate cleavage sites with the potential for ribozymatic activity

### Chemical strategies

Dolinnaya et al. (1991) reported that the condensing reagent cyanogen bromide or ethyl-3-(3′-dimethylaminopropyl) carbodiimide can be used to link two DNA strands carrying terminal 5′-hydroxyl and 3′-phosphate groups. Another chemical strategy that can achieve higher efficiency and specificity is substituting the native phosphate and hydroxyl groups at the ends of linear RNAs with other functional groups. The drawback is that the circularizing bond is not a phosphodiester bond. Several methods have been developed to enhance the purity and efficiency of the circularization reaction (Petkovic and Muller, 2015). However, intermolecular ligation products and size limitation of RNA circularization are still major downsides of chemical strategies.

### Enzymatic strategies

DNA and RNA ligases derived from T4 bacteriophage, including T4 DNA ligase 1, T4 RNA ligase 1, and T4 RNA ligase 2, are used to facilitate RNA ligation. The ligation site of T4 DNA ligase 1 needs to be located within a double-stranded region of the nucleic acid, and thus a short DNA oligonucleotide splint that hybridizes to both ends of the single-stranded RNA is needed for RNA ligation *in vitro*. One example is the circularization of a 453-nt long RNA that was successfully translated *in vitro* (Chen and Sarnow, 1995). T4 RNA ligase 1 works only on single-stranded substrates. To achieve intramolecular circularization, linear RNAs should be pre-oriented. Approximately four pre-oriented strategies are favorable for ligation with T4 RNA ligase 1. T4 RNA ligase 2 has compatible efficiencies with both double-stranded and single-stranded substrates and is much more efficient in joining nicks within double-stranded RNAs than connecting the ends of single-stranded RNAs. DNA ligase 2-induced intramolecular ligation can also be promoted by using a complementary DNA or RNA splint. RNA concatemers formed by intermolecular ligation are major competing byproducts, independent of the type of ligase used. Several strategies for minimizing intermolecular ligation byproducts have been reported (Obi and Chen, 2021). However, RNA circularization efficiency by ligases is highly dependent on the structure and length of the linear precursor.

### Ribozyme strategies

Ribonucleic acid enzymes (ribozymes) are RNA molecules that can catalyze specific biochemical reactions, acting similarly to protein enzymes. Several different types of ribozymes are used for circRNA production *in vitro*, among which, self-splicing introns are the most commonly used. Puttaraju and Been (1992) first reported that the group I intron system can be adapted to generate a circ-RNA of interest from a linear intron-containing precursor. Recently, the group I intron from the Anabaena pre-tRNA^Leu^ gene was designed and optimized for circRNA production *in vitro* (Wesselhoeft et al., 2018), leading to spontaneous circularization of the intervening exonic sequence. However, the retention of the original Anabaena exons at the ligation junction of the final circularized product was a critical limitation of this method. A serial mutagenesis and *in vitro* selection method was developed to generate modified group I introns to produce circ-RNAs without original exon sequences (Rausch et al., 2021). In addition, group II introns have also been used to generate circRNAs *in vitro*. Later, Mikheeva et al. (1997) demonstrated that a permuted group II intron from the yeast mitochondrial genome could be used for RNA circularization, but the efficiency was low. Unlike the classical group I intron permuted intron–exon (PIE) system, the group II intron PIE system allows the production of a circRNA without any extraneous sequence. A general method was recently developed to engineer the group II intron system to efficiently produce fully designed circRNAs (Chen et al., 2022). Strategies using self-splicing introns (group I and group II) generate a wide variety of circRNAs up to 5 kb in length, whereas chemical and enzymatic strategies typically show low yields for RNAs >1 kb.

In general, a reliable strategy for circ-RNA production should be highly efficient, standardized (independent of the sequence of interest, e.g. length and structure), and easy to scale up. Based on previous results, a self-splicing ribozyme strategy is a universal approach for *in vitro* circRNA production.

## Perspectives

While circRNAs have shown promise in non-human primate studies for infectious disease vaccines, clinical studies for circRNA drugs are lacking. CircRNA vaccines show less side effects and higher efficacies than mRNA vaccines, representing an ideal platform for RNA-based vaccines. However, a reliable and cost-effective manufacturing system for circRNAs has to be established to support future clinical research. For therapeutic purposes, circRNA drugs will be given at higher frequency and dosage than vaccines. Therefore, both circRNA molecules and delivery systems need to be carefully designed and optimized for specific indications.

Developing efficient and safe delivery systems will be critical for realizing the full potential of circRNA-based therapeutics. Although the current lipid nanoparticle system can deliver circRNAs into cells, its efficiency is limited and it primarily targets the liver, which hampers the potential of circRNA drugs. Thus, future development efforts could be particularly put into creating more efficient and safer delivery systems.

*[We apologize for not including those excellent works on circRNAs due to limited space. This work is partially supported by the National Natural Science Foundation of China to Y.Y. (31870814). Y.Y. is also sponsored by the Youth Innovation Promotion Association CAS, SA-SIBA Scholarship Program, and Shanghai Science and Technology Committee Rising-Star Program (19QA1410500). M.S. is an employee of CirCode Biomedicine, and Y.Y. is a co-founder of CirCode Biomedicine.]*
